# A Case of Ocular Syphilis in a 36-Year-Old HIV-Positive Male

**DOI:** 10.1155/2014/352047

**Published:** 2014-08-11

**Authors:** Amy Nguyen, Samuel Clark Berngard, Jay Patrick Lopez, Timothy C. Jenkins

**Affiliations:** ^1^University of Colorado School of Medicine, AO1, Room 8505, 12631 East 17th Avenue, Mail Stop B166, Aurora, CO 80045, USA; ^2^Department of Internal Medicine, University of Colorado School of Medicine, 12631 East 17th Avenue, B177, Aurora, CO 80045, USA; ^3^Department of Medicine and Division of Infectious Diseases, Denver Health Medical Center, 660 Bannock Street, Denver, CO 80204, USA; ^4^Division of Infectious Diseases, University of Colorado, Anschutz Medical Campus, Research Complex 2, 12700 E. 19th Avenue, Mail Stop B168, Aurora, CO 80045, USA

## Abstract

The incidence of syphilis in the United States has increased markedly over the last decade, particularly among men who have sex with men (MSM). Although uncommon, ocular involvement is a potentially devastating clinical manifestation of syphilis. Human immunodeficiency virus (HIV) infection appears to increase the risk of ocular syphilis. Because of the lack of pathognomonic features for ocular syphilis and its ability to occur in both immunocompetent and immunosuppressed individuals, prompt diagnosis requires a high index of suspicion. Ocular syphilis should therefore be considered in MSM and HIV-infected patients presenting with unexplained visual complaints. Herein, we present a case of ocular syphilis in a patient with newly diagnosed HIV.

## 1. Case Report

A 36-year-old white male presented to the emergency department complaining of seeing intermittent “floaters” over the past 3 weeks and acute, progressive vision loss over the past 4-5 days. He reported only being able to make out shadows in the left eye and blurry figures in the right eye. The patient also reported a new-onset rash around the eyes. He denied painful vision, headache, photophobia, neck stiffness, nausea, vomiting, and focal neurological deficits. Past medical history was only notable for hemolytic uremic syndrome as a child that resolved without intervention. He had used intravenous methamphetamines for the past 15 years but denied use of other illicit drugs, alcohol, and tobacco. He also endorsed having unprotected sex with numerous men in the past, although he had been in a monogamous relationship for the last 2 years.

**Figure 1:**
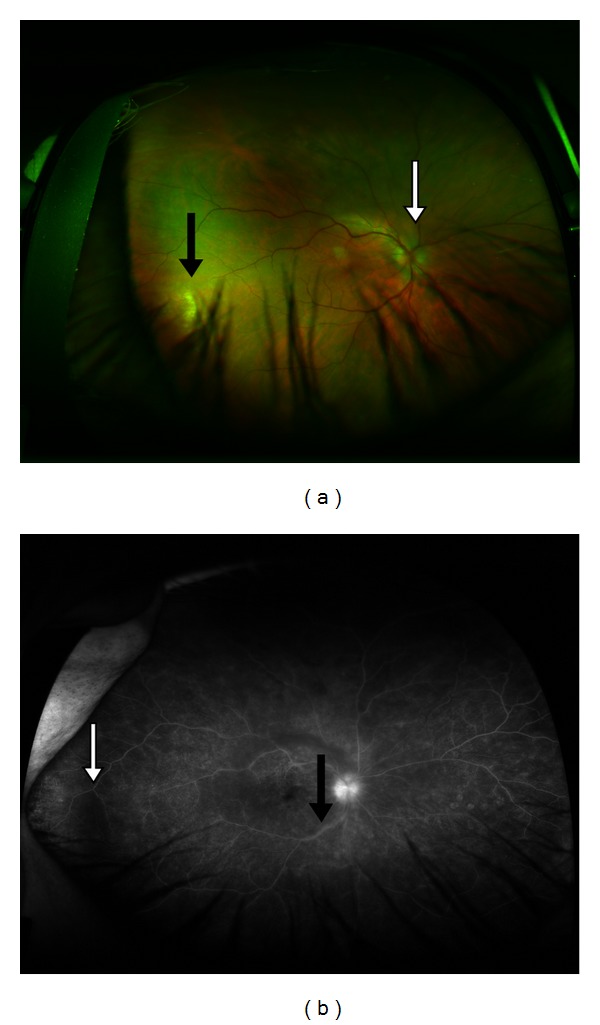
(a) Fundus photograph of the right eye demonstrates optic nerve head edema (white arrow) and white, inflammatory lesions in the retina (black arrow). (b) Fluorescein angiography of the right eye shows dye leakage (black arrow) and cellular debris in periphery of the posterior chamber (white arrow).

On physical examination, he had a bilateral periocular, maculopapular, erythematous rash with clear sclera. Visual acuity of the right eye was 20/400, but the left eye was only able to distinguish the presence of hand-waving. Fundoscopic examination revealed edema of the left optic nerve. Slit lamp examination and fluorescein angiography revealed bilateral optic nerve head edema, severe (3+/4) bilateral vitritis in all 3 chambers, extensive cellular debris, and white, inflammatory lesions in the retina (Figure 1). Also noted were bilateral posterior synechiae (adherence of the iris to the lens) creating an irregular shape to the pupils. Multiple injection sites were noted on the forearms, consistent with recent methamphetamine use.

Laboratory analysis was notable for a normal complete blood count. Lumbar puncture revealed an opening pressure of 8 cm H_2_O. Cerebrospinal fluid (CSF) analysis demonstrated 0 red blood cells/*μ*L, 11 white blood cells/*μ*L, 45 mg/dL glucose, and 35 mg/dL protein. No microorganisms were seen by Gram stain. Empiric treatment for suspected HSV infection was initiated with intravenous acyclovir.

Subsequent results of laboratory and microbiology tests included a positive HIV ELISA and confirmatory western blot. The CD4+ cell count was 377 cells/mL and the HIV RNA level was 122,480 copies/mL (log 10 = 5.1). A serum syphilis EIA screen was positive and the serum RPR titer was 1 : 256. A CSF VDRL was not performed. HSV-1, HSV-2, varicella zoster virus, and CMV PCR tests on the CSF were negative. Serum and CSF cryptococcal antigen tests were negative. The CSF bacterial culture was negative. Serum toxoplasma IgG and IgM levels were also negative.

The patient was informed of the diagnoses of HIV infection and neurosyphilis with ocular involvement. He subsequently disclosed that his current partner was HIV-positive, though the patient had never been tested for HIV. He did, however, recall being diagnosed with and treated for primary syphilis in the past. It was unknown if his partner had ever had syphilis. The patient was started on intravenous penicillin G, and both his rash and bilateral vision improved. He was discharged to complete 14 days of intravenous penicillin G. It was recommended that his partner undergoes evaluation for syphilis. Antiretroviral therapy was subsequently initiated and the patient voluntarily entered an inpatient substance abuse treatment program.

At an ophthalmology clinic visit two weeks later, the patient's vision was 20/50 bilaterally and the papilledema and uveitis had resolved. Four months after treatment, his serum RPR titer was 1 : 128. Because of the slow decline in RPR, a repeat lumbar puncture was performed approximately 6 months after treatment and revealed the CSF white blood cell count had declined to 4 cells/*μ*L and a CSF VDRL was nonreactive. Given the improved CSF pleocytosis and his continued clinical improvement, he was considered to be successfully treated. His clinical status and serum RPR titer were to be monitored on an ongoing basis.

## 2. Discussion

Syphilis is a sexually transmitted infection caused by a spirochete bacterium,* Treponema pallidum*.* T. pallidum *has the ability to infect multiple organ systems leading to protean clinical manifestations. Its natural history progresses through well-characterized stages with devastating consequences if left untreated.

The incidence of primary and secondary syphilis has increased markedly over the last decade, from 2.1 per 100,000 people in 2000 to 4.5 per 100,000 in 2011 [1, 2]. The epidemic has disproportionately affected the MSM population, while rates in women and men who have sex with women have steadily decreased. The Centers for Disease Control and Prevention (CDC) estimated that, in 2011, 72% of all primary and secondary syphilis cases occurred in MSM, which increased from just 7% in 2000 [1, 3]. The epidemic in this population is exacerbated by high rates of coexisting HIV infection and risky sexual and drug behaviors [4].

In a study of MSM presenting to sexually transmitted disease clinics, a significantly higher proportion of HIV-infected individuals had coexisting primary or secondary syphilis compared with those who were HIV-negative (10.1% versus 2.6%) [3]. The interaction between syphilis and HIV is thought to be symbiotic, though evidence for this is limited. Some studies suggest that syphilis facilitates HIV transmission by increasing expression of its CCR5 receptors or inducing expression of the HIV-1 gene in human monocytes [5]. HIV infection alters the natural history of syphilis leading to unusual and more aggressive clinical manifestations as well as earlier neurologic involvement [6].

One atypical manifestation of infection with* T. pallidum* is ocular syphilis, a form of neurosyphilis [7]. Ocular syphilis can occur at any stage of infection and may be the only clinical manifestation of infection. Although there is a wide spectrum of ocular manifestations of syphilis, uveitis is the most common [8, 9]. Clinically, patients may present with eye pain and changes in vision, including loss of visual acuity, central scotomas, and unilateral or bilateral involvement [10]. There are no pathognomonic examination findings since syphilitic uveitis appears similar to uveitis due to other etiologies. Ophthalmoscopic examination may reveal the presence of leukocytes and cloudy flares in the aqueous humor, synechiae, keratic precipitates, and other retinal lesions [11]. The differential diagnosis for ocular syphilis is broad. Since syphilis comprises less than 1-2% of all cases of uveitis, delays in diagnosis are common. The diagnosis is often not considered until a patient has failed to respond to steroid therapy [8, 12].

In HIV-infected patients, syphilitic involvement of the eye has been shown to occur earlier than in HIV-uninfected patients [6]. Furthermore, ocular syphilis in the setting of untreated HIV is more frequently bilateral and more likely to involve the posterior chamber than in those without HIV [13]. Ocular syphilis does not require immunosuppression to occur; it is therefore important to consider the diagnosis in HIV-infected patients with visual complaints regardless of CD4+ cell count.

Because* T. pallidum* cannot be cultured, diagnostic testing for syphilis typically consists of nontreponemal or treponemal serologic tests from the serum. Nontreponemal tests such as the RPR and VDRL test the reactivity of the patient's serum to a cardiolipin-cholesterol-lecithin reagent. They are nonspecific, and false-positive results are common; however, given their low cost and ease of performance, they are typically used for screening purposes. Treponemal tests include the FTA-ABS and* T. pallidum *EIA. They are more specific and nearly 100% sensitive for later-stage syphilis but are also more labor-intensive and expensive [14]. When ocular syphilis or other forms of neurosyphilis are suspected, lumbar puncture should be performed with CSF analysis in addition to serum serologic tests. Lumbar puncture serves to both confirm the diagnosis of neurosyphilis and subsequently judge the efficacy of treatment [7, 13]. A positive CSF VDRL is highly specific for neurosyphilis, although insensitive [6]. CSF examination may also reveal a lymphocytic pleocytosis or elevated protein; however, such findings may also be seen in HIV-infected patients without syphilis. In the setting of HIV, the test characteristics of both serum treponemal and nontreponemal tests may differ—likely the result of an altered immune response—either remaining positive despite initiation of therapy or reverting from positive to negative prior to successful resolution of the infection [6, 7, 15]. Furthermore, some studies have demonstrated serologic titers to be higher than expected, while others still have shown false-negatives or delayed seroreactivity [16]. Given the potential reduced sensitivity of serologic tests for syphilis in HIV-infected patients, any clinical suspicion for syphilis not supported by serologic findings warrants an attempt to visualize spirochetes microscopically [7].

Therapy for ocular syphilis is the same as for other forms of neurosyphilis. Continuous infusion of intravenous aqueous crystalline penicillin G for 10–14 days is considered first-line therapy. An alternative treatment regimen is once daily intramuscular procaine penicillin plus oral probenecid four times daily, both for 10–14 days [16]. Treatment failure in cases of ocular syphilis may occur. Therefore, continued surveillance after treatment is necessary in order to identify relapse or reinfection [6]. Historically, this has been performed through serial lumbar punctures every 6 months until the cell count has normalized, with retreatment if the CSF is not normal after 2 years [16]. More recent data suggest that serial serum RPR titers are correlated with CSF titers, predict treatment success, and may obviate the need for serial lumbar punctures [14].

Whether HIV infection affects treatment response in neurosyphilis is not clear. While the difference is likely small, HIV-infected patients may have higher rates of serologic treatment failure, further emphasizing the need for careful monitoring after treatment for relapse. In those with more advanced immunosuppression, CSF cell counts might improve more slowly. If the CSF has not normalized after 2 years, providers should consider retreatment. Despite the slight difference in treatment response, the recommended treatment regimen for HIV-positive patients remains the same as for HIV-negative patients [16].

In summary, the incidence of syphilis is increasing, particularly among MSM. The diagnosis of ocular syphilis requires a high index of suspicion and should be included in the differential diagnosis of unexplained subacute or acute visual complaints, particularly in MSM and HIV-infected patients. Rapid diagnosis and treatment are essential for good outcomes.
